# Butylated Hydroxyanisole (BHA) Disrupts Brain Signalling in Embryo–Larval Stage of Zebrafish Leading to Attention Deficit Hyperactivity Disorder (ADHD)

**DOI:** 10.3390/jox15040116

**Published:** 2025-07-09

**Authors:** Kandhasamy Veshaal, Ramasamy Vasantharekha, Usha Rani Balu, Mahesh Vallabi Aayush, Saheshnu Sai Balaji Pillai, Winkins Santosh, Barathi Seetharaman

**Affiliations:** 1 Endocrine Disruption and Reproductive Toxicology Laboratory (EDART), Department of Biotechnology, School of Bioengineering, SRM Institute of Science and Technology, Chennai 603203, Tamil Nadu, India; kk9907@srmist.edu.in (K.V.); vasanthr@srmist.edu.in (R.V.); ub3887@srmist.edu.in (U.R.B.); am5302@srmist.edu.in (M.V.A.); sb9290@srmist.edu.in (S.S.B.P.); 2 Toxicology Research on Endocrine Disruptors Laboratory (TRENDS Lab), PG & Research Department of Advanced Zoology and Biotechnology, Government Arts College, Nandanam, Chennai 600035, Tamil Nadu, India; santoshmcc@yahoo.com

**Keywords:** butylated hydroxyanisole, ADHD, zebrafish embryo, developmental toxicity, neurotoxicity

## Abstract

Background: Butylated hydroxyanisole (BHA) has been extensively used in several commercial industries as a preservative. It causes severe cellular and neurological damage affecting the developing fetus and might induce attention deficit hyperactivity disorder (ADHD). Methods: Zebrafish embryos were subjected to five distinct doses of BHA—0.5, 1, 2, 4, and 8 ppb up to 96 h post fertilization (hpf). Hatching rate, heart rate, and body malformations were assessed at 48 hpf, 72 hpf, and 48–96 hpf, respectively. After exposure, apoptotic activity, neurobehavioral evaluation, neurotransmitter assay, and antioxidant activity were assessed at 96 hpf. At 120 hpf, the expression of genes DRD4, COMT, 5-HTR1aa, and BDNF was evaluated by real-time PCR. Results: BHA exposure showed a delay in the hatching rate and a decrease in the heart rate of the embryo when compared with the control. Larvae exhibited developmental deformities such as bent spine, yolk sac, and pericardial edema. A higher density of apoptotic cells was observed in BHA-exposed larvae at 96 hpf. There was a decline in catalase (CAT), glutathione peroxidase (GPx), glutathione-S-transferase (GST), and superoxide dismutase (SOD) activity, indicating oxidative stress. There was a significant decrease in Acetylcholinesterase (AChE) activity and serotonin levels with an increase in concentration of BHA, leading to a dose-responsive increase in anxiety and impairment in memory. A significant decrease in gene expression was also observed for DRD4, COMT, 5-HTR1aa, and BDNF. Conclusions: Even at lower concentrations of BHA, zebrafish embryos suffered from developmental toxicity, anxiety, and impaired memory due to a decrease in AChE activity and serotonin levels and altered the expression of the mentioned genes.

## 1. Introduction

The study of xenobiotics, particularly synthetic antioxidants like butylated hydroxyanisole (BHA), has gained increasing attention due to their ubiquitous presence in industries such as food preservation, pharmaceuticals, and cosmetics. BHA, a mixture of 2-tert-butyl-4-hydroxyanisole and 3-tert-butyl-4-hydroxyanisole, is widely employed to prevent lipid oxidation and extend shelf life in products like oils, fats, and processed foods [1]. Despite these practical benefits, growing concerns have emerged about its safety, especially with prolonged consumption or environmental exposure with maximum concentration of 0.035 µg/L in effluents from sewage plants. When released into ecosystems, BHA can transform into tert-butylhydroquinone (TBHQ), a metabolite linked to endocrine and immune system toxicity [2]. While regulatory bodies like the FDA classify BHA as generally safe at low levels, conflicting evidence suggests it may pose risks, including carcinogenicity, cardiotoxicity, neurotoxicity, and endocrine disruption [3,4]. This duality—BHA’s utility versus its potential harm—underscores the urgent need to investigate its biological impacts, particularly on the vulnerable developmental stages. Previous research has established BHA’s capacity for both beneficial and detrimental effects. For instance, at dietary concentrations of 0.25% and 0.75%, BHA mitigates the toxicity of monocrotaline, a harmful phytochemical, by enhancing detoxifying enzyme activity, and dietary pretreatment with BHA reduced lung edema, a key indicator of phosgene-induced acute lung injury and suggest that this beneficial effect may be linked to BHA’s capacity to enhance lung tissue glutathione (GSH) levels. Conversely, higher doses (1.5–2%) are associated with tumorigenesis in the forestomach of rodents due to DNA alterations, raising safety concerns [5,6]. Beyond oncology, BHA’s neurotoxic and developmental effects are well-documented. Studies show it alters neurotransmitter levels for serotonin and norepinephrine and inhibits Acetylcholinesterase (AChE) activity, leading to impaired learning, reduced self-grooming, and diminished reflexes [7,8]. Developmentally, BHA induces body malformations, reduced heart rates, and changes in gene expression tied to the hypothalamic–pituitary–thyroid axis in zebrafish [9]. Recent investigations further highlight its role in oxidative stress and neuronal damage across models, from rats to zebrafish [10,11]. Despite this breadth of research, a critical gap persists: the specific impact of environmentally relevant BHA concentrations on neurodevelopmental disorders, like attention deficit hyperactivity disorder (ADHD), remains underexplored. ADHD, characterized by persistent inattention, hyperactivity, and impulsivity, rises from a complex interplay of genetic, neurological, and environmental factors, including disruptions in dopamine, serotonin, and AChE signalling [12,13]. While prior studies have examined BHA’s general toxicity and carcinogenic potential, or its effects on oxidative stress and neurotransmitter levels, few have linked these mechanisms to ADHD-like outcomes during early development [11]. This study addresses this gap by evaluating the effects of low-dose BHA exposure on zebrafish embryos, a model chosen for its well-conserved central nervous system (CNS) and homology to human teratological responses. Usage of the zebrafish model in research is mostly motivated by the great resemblance of its teratological and toxicological reactions to those seen in humans [14]. Zebrafish are particularly useful in neurotoxicology studies because they resemble brain structures that are homologous to components found in the human brain. The developmental processes and mechanisms of the central nervous system (CNS) in zebrafish are remarkably well conserved, making zebrafish a valuable tool for assessing neurological disorders. Therefore, the present work focuses mostly on exploring the relationships between ADHD and significant gene markers, 5HTR, COMT, BDNF, and DRD4 [15]. This study also quantifies the activity of AChE, which might be relevant for the development of ADHD. In vitro, the inhibitory action of BHA on AChE activity in isolated brain homogenates relates to Alzheimer’s disease (AD) [8].

Unlike previous work focused on broad toxicity, we investigate how BHA disrupts ADHD-specific pathways—namely, gene expression, neurobehavioral traits, and antioxidant enzyme activity at concentrations mimicking environmental exposure. Our motivation stems from the need to reconcile BHA’s widespread use with its potential to induce subtle yet significant neurodevelopmental risks, particularly in early life stages when the brain is most susceptible. By elucidating these mechanisms, this research provides novel insights into BHA’s toxicity profile and its possible contribution to ADHD-like symptoms, informing safer regulatory practices and highlighting the zebrafish model’s utility in xenobiotic studies. Thus, we hypothesize that environmentally relevant BHA levels induce ADHD-like behaviours in zebrafish via altered neurotransmission and oxidative stress. The primary aim is to clarify the intricate relationships among BHA exposure, neurotoxicity, developmental toxicity, and antioxidant responses, filling critical knowledge gaps and advancing our understanding of synthetic antioxidants’ health implications.

## 2. Materials and Methods

### 2.1. Chemicals

BHA was purchased from Tokyo Chemical Industry (Shamirpet, Telegana, India) Pvt Ltd. (CAS number: 121-00-6). The chemical was stored in a light-protected, airtight container. Five working concentrations (0.5, 1, 2, 4, and 8 ppb) were prepared by mixing BHA with 3% ethanol. These concentrations correspond to molarities of 2.77 nM, 5.55 nM, 11.1 nM, 22.2 nM, and 44.4 nM, respectively. The solvent control used was 3% ethanol. E3 media was used as the control.

### 2.2. Maintenance of Zebrafish, Embryo Collection, and BHA Exposure

Wild-type adult male and female zebrafish (Danio rerio) were acquired from a certified fish farm and housed individually in glass aquariums filled with a 2:1 mixture of reverse osmosis (RO) and tap water. The fishes were kept in a consistent environment with 14:10 h light/dark cycles at 27 °C. Before breeding, the fish were fed with goat liver and brine shrimp or flakes, and the water was changed every day. For breeding, male and female fish in a ratio of 2:1 were transferred into a breeding tank with a net at the bottom to protect the eggs released from the adult fish [16]. At the start of the cycle, spawning was induced. Eggs were collected after 1 h, washed with distilled water, and incubated in egg water until exposure. The exposure was given in six well plates with 15 embryos per well and maintained at 27 °C. The volume of exposure per well was set to 3 mL and renewed every 24 h from 4 hpf to 120 hpf, after which the larvae were kept in E3 media for further assays [17].

### 2.3. Teratological Analysis of BHA-Exposed Zebrafish Larvae

The teratological analysis performed in the BHA-treated embryo–larval stages of zebrafish was an evaluation of hatch rate, heart rate, and developmental malformations [labomed LB-210 microscope (4× magnification)]. The hatching rate of the embryos was evaluated by dividing the total number of embryos hatched by the total number of live embryos at 48 hpf. Subsequently, the heart rate of the hatched larvae was measured by counting the number of heartbeats in 60 s at 72 hpf [18]. Finally, developmental abnormalities, such as bent spine (BS), bent tail (BT), yolk sac edema (YE), and pericardial edema (PE), were examined, and the images were recorded every day until the mark of day 4 [19].

### 2.4. Evaluation of Apoptotic Activity in BHA-Exposed Zebrafish Larvae Using Acridine Orange Staining

At 5 days post-fertilization (dpf), 5 larvae were placed in 6-well plates containing 4 mL of egg water and acridine orange at a concentration of 1 μg/mL for 30 min. The larvae were then rinsed two to three times in E3 media. CMC (carboxymethyl cellulose) was used to partially immobilize the larvae, followed by microscopic examination using fluorescence microscopy (Leica DM6 Fluorescent Microscope Leica Biosystems, Nussloch, Germany) at 5× magnification to detect apoptosis (cell death) [20].

### 2.5. Neurobehavioural Assay of BHA-Exposed Zebrafish Larvae

The zebrafish embryos that were exposed to different concentrations of BHA, were subjected to neurobehavioral tests to assess stress, anxiety, and memory impairment. Stress and exploratory levels in larval zebrafish were measured using the novel tank diving test. The larvae were let to acclimatize for 2 min and the movement was tracked for 6 min. Measured behavioural parameters included the average percentage of latency to reach the upper half and the average amount of time spent in the upper half of the tank [21].

The light and dark preference test was performed to assess anxiety-like behaviour. At 5 dpf, 3 larvae from each group were subjected to a light and dark preference test performed using a 6-well plate half covered with black paper. The larval activity was video-recorded for 6 min after 2 min of acclimatization. The parameters measured were the average amount of time spent in the dark region and the average number of transitions between the light and dark sections [22].

At 6 dpf, a novel object recognition test was performed in 6-well plates, where 10 μL pipette tips labelled at the tip region were used as novel objects. Three larvae from each exposure group were acclimatized for 30 min, followed by the introduction of a novel object. The larval movements were tracked for 8 min. At 1-, 2-, and 3 h post-familiarization, the object was removed and reintroduced into the wells, and the movement was tracked for an additional 8 min. The parameters measured were the average percentage of time taken to reach the labelled end and the average amount of time spent near the labelled end [22]. All video recordings were processed by using ZebraZoom software (v1.33.18).

### 2.6. Acetylcholinesterase (AChE) Activity

The larvae were subjected to neurotransmitter assays. The homogenates from 20 larvae per concentration were used for this assay. Then, 75 µL of phosphate buffer and 25 µL of DTNB (Ellman’s reagent) were added to 25 µL of the homogenate supernatant and incubated for 5 min, after which 25 µL of acetylthiocholine iodide was added in the dark to begin the reaction. The absorbance was measured at 412 nm for 0, 30, and 60 s using Multiskan Microplate Spectrophotometer (ThermoFisher) (Waltham, MA, USA) [23].

### 2.7. Quantification of Serotonin

The serotonin quantification kit was obtained from the Bioassay Technology Laboratory (BT Bioassay: E1128Hu). The supernatant from 6 larval homogenates per concentration was used to quantify the serotonin levels following the ELISA kit protocol.

### 2.8. Antioxidant Enzyme Assay of BHA-Exposed Zebrafish Larvae

Antioxidant enzyme activities, such as Superoxide dismutase (SOD), catalase activity (CAT), glutathione peroxidase (GPx), and glutathione S transferase (GST), were performed using homogenates from 25 larvae per group in triplicates. The assays were performed in 96-well plates and absorbance was measured using the Multiskan Microplate Spectrophotometer (ThermoFisher). Protein levels were quantified using Bradford’s method with Bovine Serum Albumin as a standard. SOD activity was quantified as the percentage inhibition of epinephrine auto-oxidation using absorbance measured at 480 nm for 0, 30, and 60 s. CAT was determined through a hydrogen peroxide-based assay using absorbance measured at 405 nm. GPx activity was measured utilizing Ellman’s Reagent at an absorbance of 412 nm. GST activity was measured by adding cholro-2,4, dinitrobenzene in 99% ethanol and 20 mM reduced glutathione [24].

### 2.9. Gene Expression

The RNA was isolated from 5 zebrafish larvae per group in triplicate using the TRIzol method. The extracted RNA was quantified using NanoDrop™ (ThermoFisher) and normalized (2 µg) for cDNA synthesis. Hi-cDNA Synthesis Kit was used for cDNA synthesis (Himedia) (Thane, India).

The cDNA was synthesized and processed in quantitative Real-Time PCR to quantify mRNA copies of the genes 5HTR1aa, COMT, DRD4a, and BDNF (Table 1) using GAPDH as the housekeeping gene. The Applied Biosystems™ PowerUp™ SYBR™ Green Master Mix (Thermo Fisher Scientific, USA) (Waltham, MA, USA) was used as directed by the manufacturer for real-time PCR of the generated cDNA. The melt peak was examined to ensure proper amplification. The fold change in mRNA expression of distinct genes was calculated using 2^−ΔΔCT^ values [25].

### 2.10. Statistical Analysis

One-way Analysis of Variance (ANOVA) combined with Tukey’s multiple comparison test was performed to compare the differentiation in the data between the exposure groups and the graphs were plotted. All data was recorded as a mean ± standard deviation, while *p* < 0.05 was set as the significance level.

## 3. Result

Zebrafish embryos exposed to different concentrations of BHA such as 0.5, 1, 2, 4, and 8 ppb were subjected to various tests like teratological analysis, apoptotic activity, neurobehavioural assays, quantification of AChE activity and serotonin levels, and antioxidant enzyme assays.

### 3.1. Teratological Effects of BHA-Exposed Zebrafish Larvae

**Figure 1:**
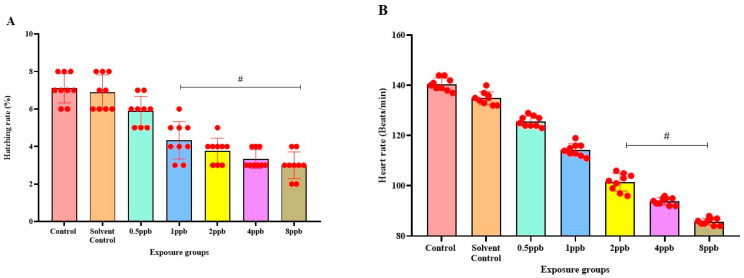
Hatching rate of zebrafish larvae at 48 hpf exposed to different concentrations of BHA, control, and solvent control. # represents *p* < 0.05 statistically significant decrease compared with control (**A**) (*n* = 5). Heart rate of zebrafish larvae at 72 hpf exposed to different concentrations of BHA, control, and solvent control. # represents *p* < 0.05 statistically significant decrease compared with control. (**B**) (*n* = 5).

**Figure 2:**
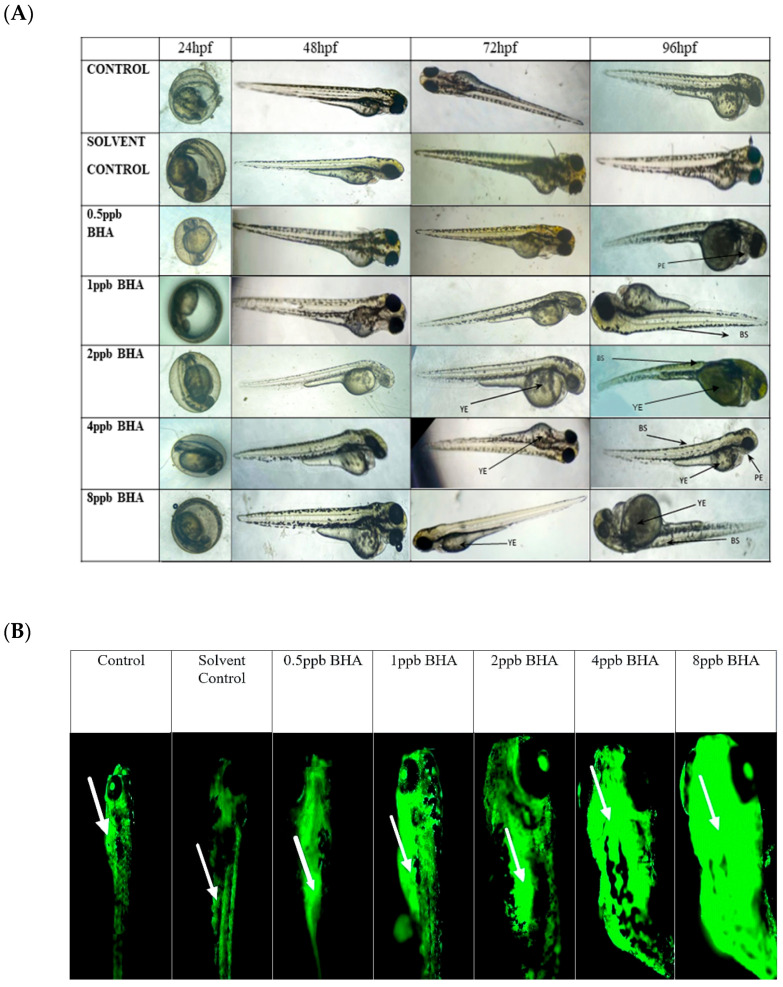
The different developmental deformities observed from 24 to 96 hpf in zebrafish larvae exposed to various concentrations of BHA, control, and solvent control (**A**). YE: yolk sac edema, BS: bent spine, PE: pericardial edema. Images of acridine orange-stained larvae showing apoptotic cells (arrows indicate regions exhibiting higher fluorescence intensity) (**B**) (*n* = 5).

The percentage of exposed embryos hatching at 48 hpf, showed a statistically significant dose-dependent decrease (*p* < 0.05) when compared with the control and solvent control from lower to higher concentration (Figure 1A). The heart rate of exposed embryos at 72 hpf, showed a statistically significant decrease in lower exposure groups, such as 4 and 8 ppb compared with control (*p* < 0.05) (Figure 1B). Developmental deformities observed from 24 to 96 hpf in BHA-treated larvae, showed the appearance of deformities from 72 hpf. The larvae exhibited physical abnormalities such as bent spine, yolk sac edema, and pericardial edema. Multiple deformities were observed in individual larvae at higher concentrations (2, 4, and 8 ppb) at 96 hpf (Figure 2A). Even after stopping the exposure, the severity and proportion of abnormalities increased over time. At 72 hpf, yolk sac edema was observed only in higher concentrations of BHA ranging from 2 to 8 ppb. Whereas, at 96 hpf, deformities were observed in higher as well as lower concentrations with multiple deformities in higher concentrations. In all exposure groups, the percentage of deformities rose with increasing duration of exposure (Table 2).

### 3.2. Evaluation of Apoptotic Activity in BHA-Exposed Zebrafish Larvae Using Acridine Orange Staining

Zebrafish larvae of control and BHA-treated groups were stained with acridine orange to identify apoptotic cells (Figure 2B). Apoptotic cell death was observed in all exposure groups except control and solvent control. A higher density of apoptotic cells was observed, with clusters of apoptotic cells present at the head, mouth, and yolk sac regions of the zebrafish larvae. Exposure groups of 4 ppb and 8 ppb were observed to have markedly higher apoptosis when compared with control and lower exposure groups.

### 3.3. Effect of BHA on Neurobehaviour in Zebrafish Larvae

**Figure 3:**
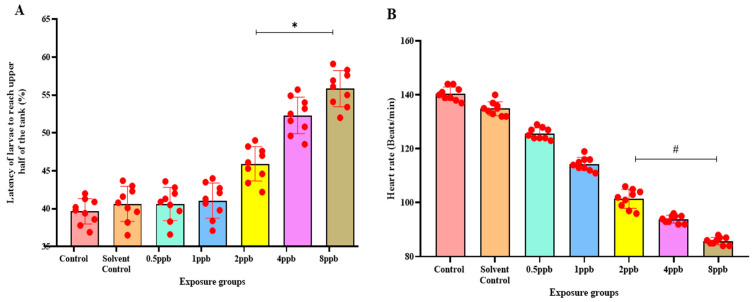

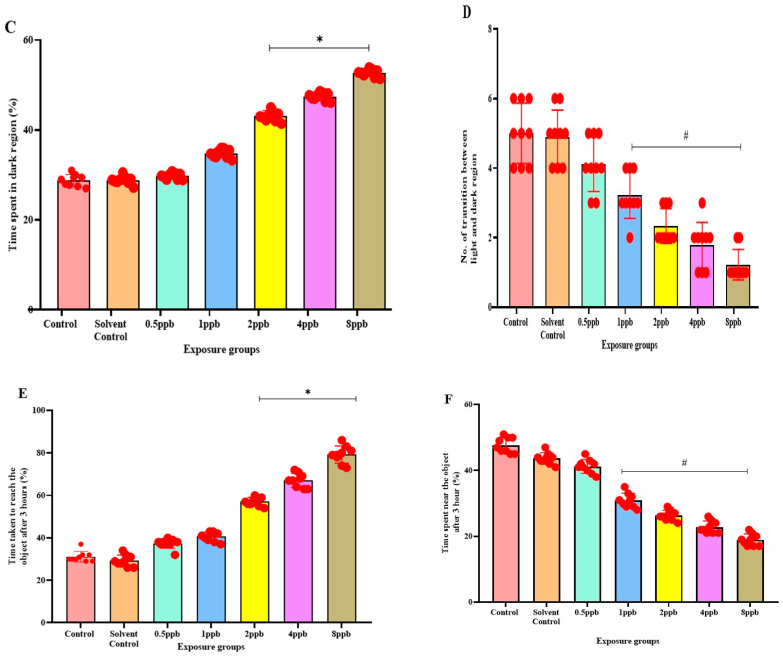
Percentage of latency to reach the upper half by zebrafish larvae exposed to different concentrations of BHA, control, and solvent control. * represents *p* < 0.05 statistically significant increase compared with control. (**A**) (*n* = 5). Percentage of time spent in the upper half by zebrafish larvae exposed to different concentrations of BHA, control, and solvent control. # represents *p* < 0.05 statistically significant decrease compared with control (**B**) (*n* = 5). Percentage of time spent in the dark region by zebrafish larvae exposed to different concentrations of BHA, control, and solvent control. * represents *p* < 0.05 statistically significant increase compared with control (**C**) (*n* = 5). Number of transitions from light to dark by zebrafish larvae exposed to different concentrations of BHA, control, and solvent control. # represents *p* < 0.05 statistically significant decrease compared with control (**D**) (*n* = 5). Percentage of time taken to reach the object in 3 h by zebrafish larvae exposed to different concentrations of BHA, control, and solvent control. * represents *p* < 0.05 statistically significant increase compared with control (**E**) (*n* = 5). Percentage of time spent near the object in 3 h by zebrafish larvae exposed to different concentrations of BHA, control, and solvent control. # represents *p* < 0.05 statistically significant decrease compared with control (**F**) (*n* = 5).

The novel tank diving test showed that when compared with the control, higher concentrations such as 2, 4, and 8 ppb BHA exposure groups demonstrated a statistically significant increase in the percentage of latency to reach the upper half (Figure 3A). The amount of time spent in the upper half also decreased significantly from 1 ppb to 8 ppb of BHA (Figure 3B).

In the light and dark preference test, the exposure groups, i.e., 2, 4, and 8 ppb of BHA, exhibited a statistically significant dose-dependent increase in time spent in the dark region when compared with the control groups (Figure 3C). The number of transitions from the light to dark region was also decreased in all the BHA exposure groups with a significant decrease from 1 to 8 ppb (Figure 3D). Therefore, the results indicate that BHA exposure induced increased anxiety-like behaviour in a dose-dependent fashion.

In the novel object recognition test on time increment basis, the time taken to reach the novel object increased, indicating reduced recognition in a dose-dependent manner across all exposure groups. A significant increase was observed in 2 to 8 ppb in the third hour (Figure 3E) compared with control groups. With an increase in time, the exposed larvae spent less time near the novel object. A significant dose-dependent increase from 1 to 8 ppb in the third hour (Figure 3F) was observed.

### 3.4. Effect of BHA on Acetylcholinesterase Activity in Zebrafish Larvae

**Figure 4:**
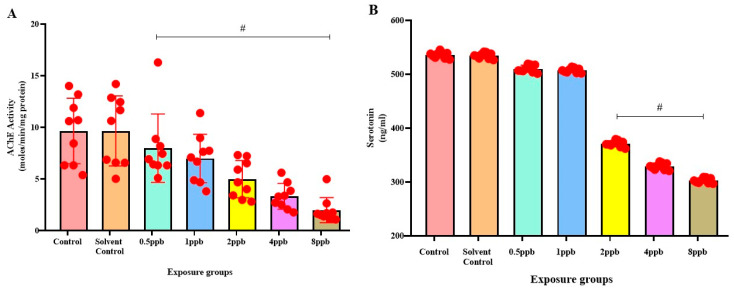
Acetylcholinesterase activity in zebrafish larvae exposed to different concentrations of BHA, control, and solvent control. # represents *p* < 0.05 statistically significant decrease compared with control (**A**) (*n* = 5). Serotonin levels in zebrafish larvae exposed to different concentrations of BHA, control, and solvent control. # represents *p* < 0.05 statistically significant decrease compared with control (**B**) (*n* = 5).

AChE activity was found to be the highest in the control and solvent control groups, with no significant difference between them. The BHA-exposed groups exhibited significant dose-dependent decrease in AChE activity when compared with control (Figure 4A).

### 3.5. Effect of BHA on Serotonin Levels in Zebrafish Larvae

The control group was found to exhibit the highest level of serotonin, with no significant difference compared with solvent control and BHA-exposed groups of 0.5 and 1 ppb. A considerable dose-dependent decline in the levels of serotonin was observed in 2, 4, and 8 ppb BHA-exposed groups when compared with control groups (Figure 4B).

### 3.6. Effect of BHA Exposure on Antioxidant Enzymes in Zebrafish Larvae

**Figure 5:**
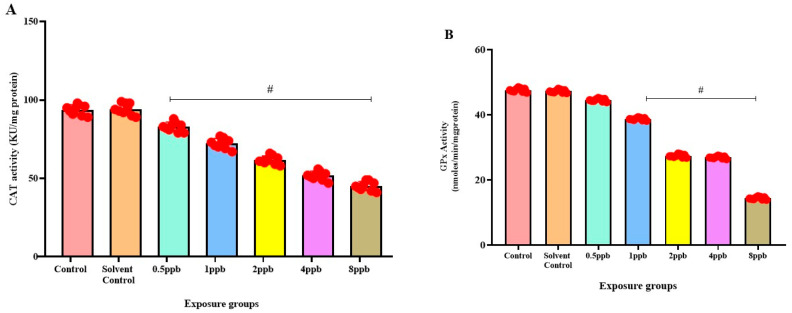

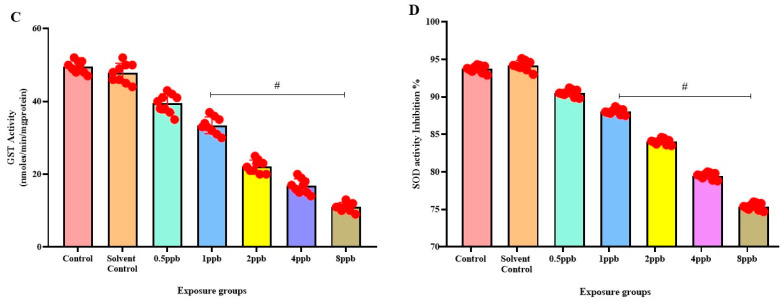
Activity of Catalase enzyme in exposed zebrafish larvae that were exposed to different concentrations of BHA, control, and solvent control. # represents *p* < 0.05 statistically significant decrease compared with control (**A**) (*n* = 5). Activity of glutathione peroxidase enzyme in exposed zebrafish larvae that were exposed to different concentrations of BHA, control, and solvent control. # represents *p* < 0.05 statistically significant decrease compared with control (**B**) (*n* = 5). Activity of glutathione-S-transferase enzyme in exposed zebrafish larvae that were exposed to different concentrations of BHA, control, and solvent control. # represents *p* < 0.05 statistically significant decrease compared with control (**C**) (*n* = 5). Inhibition % of Superoxide dismutase enzyme in exposed zebrafish larvae that were exposed to different concentrations of BHA, control, and solvent control. # represents *p* < 0.05 statistically significant decrease compared with control (**D**) (*n* = 5).

In comparison to the control, there was a significant dose-dependent reduction in CAT activity in all BHA-exposed groups (0.5 to 8 ppb) (Figure 5A). Similarly, a dose-dependent decrease in GPx activity was observed in all BHA-exposed groups with significant differences in 1, 2, 4, and 8 ppb BHA compared with control groups (Figure 5B). A significant dose-dependent decrease was observed in GST activity in all BHA-exposed groups (1 to 8 ppb) compared with control groups (Figure 5C). A significant dose-dependent decrease in SOD activity in all BHA-exposed groups (1 to 8 ppb) was observed compared with the control groups (Figure 5D).

### 3.7. Effect of BHA on the Expression of ADHD-Related Genes

**Figure 6:**
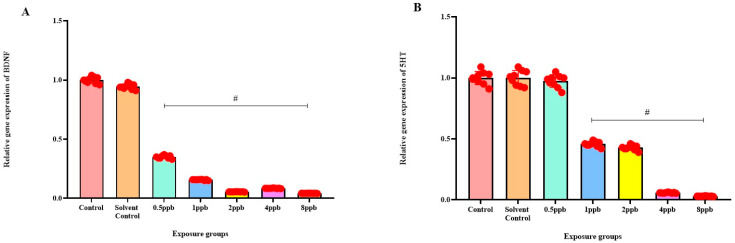

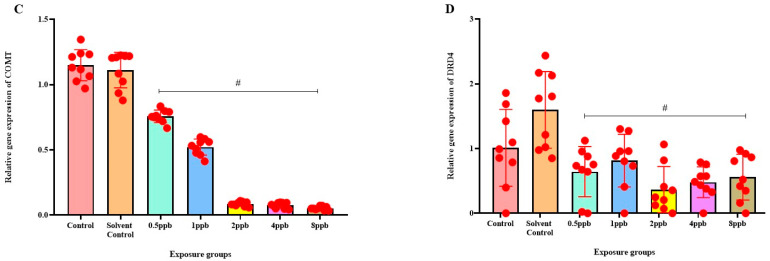
Expression of BDNF in zebrafish larvae exposed to different concentrations of BHA, control, and solvent control. # represents *p* < 0.05 statistically significant decrease compared with control (**A**) (*n* = 5). Expression of 5 HT in zebrafish larvae exposed to different concentrations of BHA, control, and solvent control. # represents *p* < 0.05 statistically significant decrease compared with control (**B**) (*n* = 5). Expression of COMT in zebrafish larvae that were exposed to different concentrations of BHA, control, and solvent control. # represents *p* < 0.05 statistically significant decrease compared with control (**C**) (*n* = 5). Expression of DRD4 in zebrafish larvae exposed to different concentrations of BHA, control, and solvent control. # represents *p* < 0.05 statistically significant decrease compared with control (**D**) (*n* = 5).

Levels of BDNF expression showed a statistically significant dose-dependent decrease across all exposure groups from 0.5 to 8 ppb BHA compared with the control groups (Figure 6A). The 5 HT activity in zebrafish larvae exposed to BHA, showed a statistically significant decrease across all exposure groups from 1 to 8 ppb when compared with the control groups (Figure 6B). A significant dose-dependent decline in COMT expression level was noted across all exposure groups from 0.5 to 8 ppb BHA compared with control groups (Figure 6C). A significant decrease in DRD4 activity was observed in zebrafish larvae exposed to BHA across all exposure groups from 0.5 to 8 ppb when compared with the control groups (Figure 6D).

## 4. Discussion

BHA, a prominent antioxidant denoted as E320, has been widely employed as a preservative in diverse products, encompassing food, cosmetics, and pharmaceuticals. Despite it being generally acknowledged as safe, BHA possesses the ability to transform into chromatic metabolites resembling tertiary butyl hydroquinone (TBHQ), the ramifications of which, maybe more harmful than those associated with BHA itself [30]. Elevated concentrations of BHA exposure can induce teratogenic distortions, neurobehavioral deficits, and anomalies in the activity of antioxidant enzymes and neurotransmission in larval zebrafish. The principal aim of behavioural neurobiology is to delineate the genes and neural circuits governing the behaviour across organisms. Currently, zebrafish larvae benefit from a variety of behavioural assays encompassing sensorimotor and cognitive dimensions [31]. Two critical endpoints in early-life stage zebrafish larvae testing are hatching and heart rate. The present study discovers that at 48 hpf, embryos exposed to BHA exhibited a delay in hatching and a decrease in heart rate compared with the control groups.

The impact of BHA exposure on the dislodgement of the chorionic membrane is implicated in delayed hatching. Reduced heart rate has been attributed to the effect of AChE inhibitors. BHA, a proven AChE inhibitor from the present study, can thus be attributed to the measured reduction in heart rate [32]. High concentrations of BHA exposure in the present study increased the percentage and likelihood of deformities, including bent tail and spine, and yolk sac and pericardial edema. Our study shows that BHA affects embryonic development, causes deformities, and impairs cognitive processes, as observed in neurobehavioral assays. The novel tank diving test was used to assess the anxiety through swimming behaviour. This evaluation unveiled an escalated manifestation of anxiety-like behaviour in larval zebrafish, exposed to higher concentrations of BHA when introduced into a novel environment. Increased exploratory behaviour and reduced immobility time observed in this test can also be correlated to hyperactivity symptoms as observed in ADHD [25].

The light and dark preference test involves evaluating the behaviour of zebrafish larvae exposed to BHA during alternating light and dark periods. Recent observations substantiate the outcomes of the light and dark preference test, revealing that high concentrations of BHA-exposed larval zebrafish tend to spend a smaller duration in the dark region. This behaviour signifies heightened stress levels, increased anxiety, and a reduction in exploratory tendencies. An analogous study suggested that the nature of the dark avoidance is an anxiety-like attribute in youthful juvenile zebrafish larvae [33]. BHA exposure reduces the memory- and recognition-associated sections of cognition. In the novel object recognition test (NOR), the larvae took a significantly longer time to reach the object and also spent less time near it, indicating recognition impairment and poor memory, respectively [34]. Additionally, they spent significantly less time near the new object, indicating reduced memory compared with the control groups at different time intervals. An analogous trend was observed in separate studies for mice exposed to toluene and female mice exposed to BHA. The exposed mice could not separate or differentiate between the new object and the familiar object, which was set up to be dependent on the hippocampal region [35].

Similar investigations were conducted with chlorpyrifos and atrazine on common carp, where chlorpyrifos, acting on the central nervous system, served as an inhibitor of AChE, changing the behavioural system and resulting in heightened locomotor activity [36]. At the neuromuscular junction, the enzyme AChE catalyzes the breakdown of the neurotransmitter acetylcholine (ACh). In zebrafish larvae exposed to BHA, AChE activity showed a pronounced reduction across all exposure levels, implying that increased reactive oxygen species (ROS) production is likely causing neuronal damage in cholinergic neurons. A reduction in exertion would result in a decreased breakdown of ACh [37]. As a result of the accumulation of ACh in synapses and the continual activation of cholinergic receptors, synapses become desensitized. Neurons in the brain system, such as the PFC, mediate attention through the reception of inputs from sensory association areas. In this study, AChE activity was used as an indication of ADHD activity. A decrease in AChE activity due to exposure to BHA results in inhibition of neuronal signal transduction between neurons, in turn impairing brain structures in the PFC that mediate attention and memory. Alterations in exertion of AChE might be associated with heightened oxidative stress induced by BHA, leading to the observed anxiety in this study. Serotonin exertion revealed a notable decrease in serotonin levels within the exposure groups, implying a state of depression among the exposed larvae.

Distinctive variances specific to agents were noted in terms of growth, anxiety-like behaviour, and the corticoid response to novelty during glucocorticoid treatment [38]. Zebrafish larvae and humans exhibit comparable apoptotic mechanisms. Studies conducted in vitro have indicated that the inhibition of caspase peptides can effectively prevent the fragmentation of DNA in zebrafish larvae [20]. The neurobehavioral phenotypes identified in the immunofluorescence assay are likely influenced by apoptotic clusters in the hippocampus, a brain region governing attention and memory. These clusters manifest as dark-spotted patches in the head region, contributing to oxidative stress and neuroinflammation. When the ROS of the body, such as superoxide and peroxide, along with antioxidant levels, are not in equilibrium, it results in oxidative stress [39]. The hydroxyl groups at the double bond of ascorbic acid act as reducing agents that are highly reactive, with ROS ultimately being oxidized to stable ascorbic ions [40]. SOD plays an important role in the body’s antioxidant defence as the sole enzyme in its group that defends against superoxide radicals. The mechanism of action of SOD is the catalysis of superoxide radicals into oxygen and hydrogen peroxide [41]. CAT is responsible for the conversion of hydrogen peroxide, a product of SOD action, into water and oxygen. In our study, when compared with the control, there was a significant dose-dependent decrease in catalase activity in all BHA-exposed groups. This reduction in CAT activity is likely due to the denaturation of the enzyme caused by elevated levels of reactive oxygen species (ROS).

GST facilitates the conjugation of reduced glutathione with xenobiotic substrates, allowing faster xenobiotic concurrence. GST acts as a safeguard against ROS and lipid peroxidation when conjugated with reduced GSH. A significant decrease was observed in GST activity, lowering the cell’s detoxification and excretion process. SOD activity would be stimulated to maintain ROS levels in tissues at a consistent level. GPx is a catalytic enzyme that catalyzes the reaction of glutathione (GSH) with H_2_O_2_ and other organic peroxides, playing a pivotal role in cellular detoxification [42]. In the current study, it is observed that BHA reduces the inhibition by SOD and the specific activity of CAT, GST, and GPx in zebrafish larvae. Alteration of memory and recognition, induction of anxiety, stress-like behaviour, and depression were also observed in larval zebrafish. BHA has been shown to cause a decrease in AChE activity by causing impairment in the neuronal signal transduction across neuronal synapses, negatively affecting brain function.

The PFC, an important brain region responsible for mediating attention towards incoming stimuli and filtering out irrelevant sensory inputs, was affected by a decrease in AChE, leading to its impairment. Damage to the PFC in the form of lesions in monkeys has been shown to lead to ADHD-like symptoms, like those observed in the neurobehavioral assay performed on the zebrafish larvae [43]. A significant decrease in serotonin levels was observed across all exposure groups in the study, suggesting that BHA exposure may lead to depressive effects by disrupting the serotonergic signalling pathway. The decline in serotonin levels can cause different neurological and behavioural changes, including mood disturbances, altered stress responses, and symptoms resembling ADHD, i.e., increased hyperactivity and aggression, given serotonin’s important role in regulating mood, behaviour, and neurological functions [44]. The activity of 5-HT is different in individuals with ADHD compared with those without it. It plays a role in alternating the cognitive processing style observed in people with ADHD [15].

Brain-derived neurotrophic factor (BDNF) is crucial for the growth, differentiation, and survival of neurons. It plays a significant role in synaptic plasticity and cognitive functions, which are critical during neurodevelopment. BDNF expression levels exhibited a statistically significant dose-dependent decrease. Reduced BDNF levels can impair these processes, potentially leading to neurodevelopmental disorders like ADHD [45]. Similarly, a decrease in COMT level suggests a disruption in the inactivation of dopamine within the PFC, potentially leading to neurodevelopmental conditions like ADHD and obsessive–compulsive disorder (OCD), as changes in the COMT gene have been associated with these disorders [46]. The disturbance in dopamine regulation within the PFC could contribute to the development of such conditions as COMT has been considered especially relevant in regulating synaptic dopamine concentration in the PFC because of the interference of the Val/Met functional SNP [47]. Furthermore, a decline in DRD4 levels, responsible for regulating attention and behaviour, may indicate a disruption in the brain regions governing these functions, potentially leading to symptoms associated with ADHD. This disruption in dopamine signalling pathways, influenced by variations in DRD4, could contribute to behavioural changes and attention deficits observed in affected individuals. In a study, researchers conducted population association studies and family-based analyses, finding a significant association between certain alleles of the DRD4 gene and ADHD. These findings provide evidence supporting the link between the DRD4 gene and a refined phenotype of ADHD [48].

## 5. Conclusions

The effects of BHA on the human body are currently being thoroughly discussed, as BHA is considered to be safe for intake in trace quantities by regulatory administrations such as the FDA. To address uncertainties about how BHA affects the cholinergic and serotonergic systems, we utilized zebrafish larvae as a model, commonly employed in toxicity investigations. The presence of BHA caused morphological teratogenic abnormalities in zebrafish larvae, including delayed disruption of the chorionic membrane, which leads to reduced birth rates and a higher density of apoptotic cells that were found in the head, mouth, and yolk sac region of the larvae, as observed in the present study. It was also noted that acute BHA exposure was linked with reduced heart rate in the model subject, hinting towards a negative impact on cardiac function, which leads to a slowdown in embryonic heart rate. High stress and anxiety were exhibited in the zebrafish larvae that were exposed to BHA according to the novel tank diving test and the light and dark preference test. Recognition and memory were also affected, as observed in the object recognition test, which could also mean that the serotonin pathways have been compromised due to BHA toxicity. A decline in serotonin levels in the exposure groups also indicates that the serotonergic pathways have been altered due to an excess of the antioxidant enzymes induced by BHA exposure. The BHA exposure groups showed a sharp decline in the AChE activity in the larvae compared with the control groups, which hints towards an increase in ROS production, which causes neuronal injury in cholinergic neurons. This could inevitably lead to impaired neuromuscular function, sensory processing deficits, and feeding abnormalities, thus, affecting social interaction and altering breeding patterns. The exhibition of ADHD-like symptoms, as a result of BHA exposure in zebrafish larvae and its possible parallel effects on humans through the consumption of this preservative in food products, is of major concern. This emphasizes careful regulation of BHA usage, particularly in the food industry. Moreover, further research may be beneficial in altering industry perceptions of BHA as a safe preservative, potentially encouraging more prudent utilization.

## Figures and Tables

**Table 1:** List of gene-specific primers used for real-time PCR.

Genes	Primer (5′–3′)	Gene ID	Reference
5-HTR1aa	Forward Primer: ATCTCTCTAGACGTGCTGTGCTGC Reverse Primer: GTTTTCCTTATGCGAAACCTCGCC	100001828	[26]
COMT	Forward Primer: ACTCGACCACAGCGTCTGCT Reverse Primer: AGCCCATTCGCGGTGTCTGC	565370	[27]
DRD4a	Forward Primer: ATGGTAGAGGCAGACATGCCA Reverse Primer: TTAGCATGCTCAGGCTAGCAG	503564	[28]
BDNF	Forward Primer: ATAGTAACGAACAGGATGG Reverse Primer: GCTCAGTCATGGGAGTCC	58118	[29]

**Table 2:** Percentage of developmental deformities observed from 24 to 96 hpf in zebrafish larvae exposed to different concentrations of BHA, control, and solvent control. Twenty larvae from each group were assessed for deformities.

Exposure Groups	24 hpf	48 hpf	72 hpf		96 hpf		
			BS	YE	BS	PE	YE
Control	N/O	N/O	N/O	N/O	N/O	N/O	N/O
Solvent Control	N/O	N/O	N/O	N/O	N/O	N/O	N/O
0.5 ppb BHA	N/O	N/O	N/O	N/O	N/O	15%	N/O
1 ppb BHA	N/O	N/O	N/O	N/O	20%	N/O	N/O
2 ppb BHA	N/O	N/O	N/O	25%	15%	N/O	25%
4 ppb BHA	N/O	N/O	N/O	30%	45%	15%	30%
8 ppb BHA	N/O	N/O	N/O	35%	50%	N/O	40%

YE: yolk sac edema, BS: bent spine, PE: pericardial edema. N/O = not observed.

## Data Availability

The original contributions presented in this study are included in the article. Further inquiries can be directed to the corresponding author.

## Abbreviations

BHA: butylated hydroxyanisole; ADHD: attention deficit hyperactivity disorder; AChE: Acetylcholinesterase; AD: Alzheimer’s disease; hpf: hours post-fertilization; dpf: days post-fertilization; NTD: novel tank diving; ROS: reactive oxygen species; SOD: superoxide dismutase; CAT: catalase; GSH: glutathione; MDA: malondialdehyde; SD: standard deviation.
